# Combined Functional Electrical and Transcranial Direct Current Stimulation for Foot Drop: A Case Series

**DOI:** 10.14740/jmc5333

**Published:** 2026-07-28

**Authors:** Sylvester Carter, Gregory Thielman

**Affiliations:** a Department of Physical Therapy, Saint Joseph’s University, Philadelphia, PA, USA; b Patricia Leahy Movement Science Lab, Saint Joseph’s University School of Health Professions, Philadelphia, PA, USA

**Keywords:** Falls, Foot drop, Minimum toe clearance, Functional electrical stimulation, Case series, Transcranial direct current stimulation, Stroke

## Abstract

Stroke is a leading cause of severe long-term disability, and foot drop is a common post-stroke gait impairment that may reduce toe clearance, increasing the risk of toe contacts and, in turn, trip-related falls. This case series evaluated the novel combination of transcranial direct current stimulation (tDCS), functional electrical stimulation (FES) to the tibialis anterior, and physical therapy training to improve minimum toe clearance (MTC) in individuals with chronic stroke. The rationale for this combined approach was that tDCS may prime the motor cortex and enhance neuroplastic responsiveness, while FES provides task-specific peripheral stimulation to the dorsiflexors during gait-related training, potentially increasing MTC and minimizing toe drag. Four individuals with chronic stroke participated in an 8-week treatment program with sessions twice a week. Assessments were conducted at baseline, 4 weeks, and 8 weeks. Participant 1 started with tDCS alone for 4 weeks, then added FES for the remaining 4 weeks. Participant 2 began with FES for the first 4 weeks, and tDCS was added for the remaining 4 weeks. Participants 3 and 4 received both tDCS and FES throughout the entire 8-week program. Participants 1 (58-year-old female) and 4 (42-year-old male) showed no active ankle dorsiflexion at baseline, while participants 2 (66-year-old male) and 3 (36-year-old male) had a grade of 2 out of 5 for ankle dorsiflexion strength at baseline. Toe clearance and functional outcomes were collected at each assessment. Participants 1 and 4 demonstrated no meaningful change in MTC, whereas participants 2 and 3 both showed modest improvements. Among these responders, MTC increased by an average of 0.63 cm. These findings suggest that combining cortical neuromodulation with localized FES and physical therapy may improve toe clearance in select individuals with chronic stroke. However, because tibialis anterior activation was not directly measured, it remains unclear whether the observed changes were due to improved dorsiflexor activation or other compensatory gait strategies.

## Introduction

Approximately 795,000 people experience a stroke in the United States annually [1]. Foot drop is one of the most common disabilities experienced by stroke survivors, affecting between 20% and 30% of individuals [2, 3]. The consequences of foot drop include walking speed reductions [4], instability during walking [5], and increased risk of toe contacts due to a plantarflexed foot [6], all of which may increase fall risk [6–14]. In fact, in persons more than 1 year after a stroke, 36% experienced a fall within the previous year compared to 24% for age- and gender-matched controls [15]. The consequences of falls can include cuts and bruises, fractures, fear, a high caregiver burden, increased health services utilization, impaired quality of life, and death [16–18].

Minimum toe clearance (MTC) has been evaluated as a means of assessing the risk of trip-related falls [19–21]. Interest in this variable arose because foot drop increases ankle plantarflexion [22, 23], which limits swing-phase limb shortening [24–26], bringing the foot closer to the ground, increasing the risk of trip-related falls [6, 21, 27, 28]. Data from community-dwelling individuals with stroke show that 7% of falls and 22% of near falls are linked to difficulty clearing the affected limb, especially during the swing phase for near falls (19.4%) [29, 30]. Additionally, ankle plantarflexion at toe-off is approximately 1° greater during trip steps than during non-trip steps in individuals with chronic stroke [25].

Methods to address foot drop include ankle-foot orthoses (AFOs), surgical options, medical approaches, and functional electrical stimulation (FES), with no single best treatment identified [31]. Many methods have limited patient acceptance and high cost [31]. Among these, FES has the highest-quality evidence supporting its effectiveness, despite the small sample sizes of existing studies [31]. FES has been shown to improve walking speed, active dorsiflexion, balance, and daily activities, especially when combined with physical therapy training (PTT) [32, 33]. The hypothesized mechanisms underlying these improvements include increased muscle strength, improved joint flexibility, and cortical reorganization facilitated by volitional intent during stimulation [34]. However, one study found that after 42 weeks of FES, 32% of participants did not demonstrate a clinically meaningful improvement in gait speed, and 45% did not demonstrate a meaningful improvement in functional gait ability [3]. These findings highlight the need for adjunct therapies to enhance neuromuscular responses.

Transcranial direct current stimulation (tDCS) shows promise for reducing fall risk in persons post-stroke (PPS), despite limited research [35]. This non-invasive method increases cortical excitability with anodal stimulation and decreases it with cathodal stimulation [36]. Additionally, tDCS can induce neuroplastic changes in the motor cortex and enhance motor performance through “priming,” which may help balance transcallosal inhibition between hemispheres by increasing excitability in the ipsilesional hemisphere and decreasing excitability in the contralesional hemisphere [37]. Although tDCS lacks specificity for the lower limb because the lower-limb representation is located on the medial aspect of the motor cortex, it has been linked to reduced fall risk and improved lower-limb function in PPS [38]. Improvements have also been observed on tests such as the 6-min walk test and Tinetti’s performance-oriented mobility assessment, as well as in specific gait metrics, including gait speed and temporospatial measures [39, 40]. Additionally, anodal stimulation can enhance excitability in corticospinal projections to the tibialis anterior muscle, with effects lasting up to 60 min [41].

Evidence from animal studies suggests that pairing peripheral nerve stimulation (e.g., FES) with cortical stimulation (e.g., tDCS) may offer additional benefits [37]. However, results from human studies are mixed [42, 43]. One study found that FES combined with tDCS improved upper-limb function in PPS [42], whereas another found no significant effect on tibialis anterior muscle activity or static balance when FES was applied in isolation rather than during physical therapy [43]. This distinction is important because FES combined with physical therapy appears to yield better outcomes [32]. Therefore, this case series aimed to assess whether combining FES and tDCS during a physical therapy session could reduce foot drop by improving toe clearance and ankle dorsiflexion in PPS.

## Case Report

### Investigations

Table 1 presents the demographic information of each participant. This case series investigation received expedited IRB approval prior to enrollment of the reported participants. Participant 1, a 58-year-old female, had a right-sided cerebrovascular accident (CVA) that resulted in left lower limb weakness 13 years before the intervention. The participant ambulated without an AFO and could not produce active dorsiflexion outside of a synergistic flexion pattern with excessive ankle inversion on the affected left side. Neither participant 2 (a 66-year-old male) nor 3 (a 36-year-old male) ambulated in the community with their AFOs, but could produce active dorsiflexion, with a manual muscle test strength of grade 2. Participant 4 (a 42-year-old male) ambulated using a hinged AFO with a dorsiflexion stop and could not produce active dorsiflexion.

**Table 1:** Patient Characteristics

Subject Number	Year of stroke	Affected side (L/R)	Ethnicity	Age (years)	Sex (M/F)	Weight (kg)	Height (m)	Past medical history
1	2010	L	Black/African American	58	F	68.04	1.75	Bowel and bladder irregularities, allergies, vascular clots
2	2007	L	Black/African American	66	M	105.91	1.69	Hypertension, diabetes
3	2020	L	Black/African American	36	M	68.49	1.69	Nerve disease/disorder, speech/communication deficits, paresthesia
4	2022	R	Black/African American	42	M	92.99	1.68	

L: left; R: right; M: male; F: female; m: meters; kg: kilograms.

### Diagnoses

Outcome measures included kinematic motion capture, functional gait assessment (FGA), Activity-specific Balance Confidence Scale (ABC), Walk-12, and the Stroke Impact Scale.

For the motion capture, participants were fitted with a modified Cleveland Clinic marker set, in which four clusters of four markers were attached to the thighs and shanks, and wand-mounted markers were also attached bilaterally to the anterior superior iliac spines (ASIS) and the sacrum. Additionally, seven 14 mm reflective markers were attached to the shoe at the upper ridge of the posterior surface of the calcaneus, sustentaculum tali, and lateral aspect of the calcaneus (peroneal tubercle), and the heads of the first, second, and fifth metatarsal (MET) and at the tip of the shoe/second digit if barefooted. In Visual 3D (C-Motion, Maryland, USA), a single-segment foot was built with the first and fifth MET head markers and a virtual ankle joint (constructed from virtual markers of the lateral and medial malleoli) using the calibrated anatomical system technique (CAST) described by Cappozzo et al [44, 45].

During each testing session, all participants completed 25 walking trials, each at a self-selected normal walking speed. All participants walked without an AFO, either barefoot (participant 1) or in their shoes (participants 2, 3, and 4). Eight infrared cameras (Bonita 10 cameras; VICON Nexus software v. 2.15.0; Oxford, UK) recorded at 100 Hz and captured the marker trajectories in the central 7 m of a 13-m walkway. Participants began walking at least 2.5 m before the cameras’ collection volume to reach steady-state walking in the volume.

#### Data analysis

The marker data were initially processed using Vicon Nexus 15 software (Oxford, UK), during which marker labeling and gap filling were performed. In Visual 3D (C-Motion, Maryland, USA), marker trajectories were filtered with a zero-phase shift, low-pass, fourth-order Butterworth filter with a cutoff frequency of 6 Hz. To determine the joint angles, 3D marker trajectories were processed with Visual 3D pipelines using the Cardan rotation sequence X-Y-Z to derive joint angle data. For flexion-extension movements in the sagittal plane, rotations were characterized around the mediolateral (X) axis. Hip adduction and abduction movements were negated on the left limb, allowing for interpretation similar to the right limb. The gait events, heel strike, and toe-off were identified using kinematic data with an automated program in Visual 3D [46]. All events were verified by visual inspection. Next, Visual 3D’s metric compute temporal distance command was used to calculate temporal distance gait parameters from the gait events.

**Figure 1:**
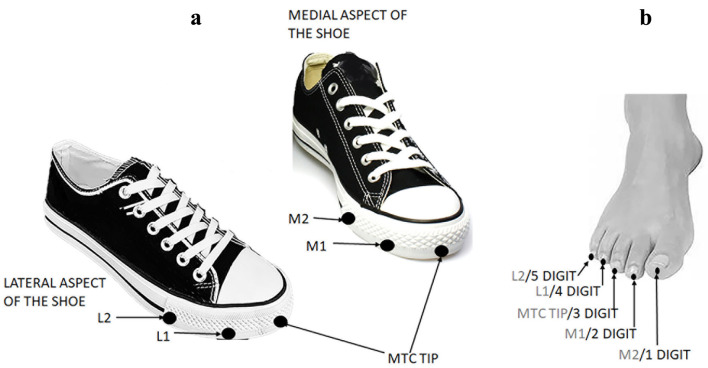
The location of virtual points on the shoe (a) and barefoot (b).

Visual 3D’s (C-Motion, Maryland, USA) Landmark function was used to define the virtual points. A digitizing pointer was used during the static calibration trial to create the virtual markers (landmarks) and to establish their relationship to the local coordinate system of the tracking cluster (first and fifth MET and shoe tip/second digit-tip markers) [47–50]. The virtual points comprised a point on the tip of the shoe and four other virtual points on the shoe’s medial, anterior, and lateral aspects, except when barefoot, when the tips of each digit comprised the virtual points (Fig. 1). These points are positioned to characterize the front of the shoe because this is the part that may contact the floor in the mid-swing phase of the gait cycle and result in a trip-related fall [51].

### Treatment

The interventional procedures were designed to provide preliminary, individual level data on whether adding tDCS or FES would yield further improvements beyond either modality alone. Since either intervention may affect MTC independently, performing tDCS and FES together at the outset would have precluded our ability to determine whether adding the other modality was associated with improvements beyond either modality alone. Therefore, for this exploratory evaluation, participant 1 began the first 4 weeks with tDCS alone, then added FES at week 4. This sequence allowed us to examine whether the addition of FES was associated with further change beyond that observed during the initial tDCS only phase. Conversely, participant 2 began the first 4 weeks with FES alone and added tDCS at week 4, allowing us to examine whether the addition of tDCS was associated with further change beyond that observed during the initial FES only phase. Next, we examined whether the combined treatment of tDCS and FES throughout the entire 8-week intervention period was associated with improvements in MTC in participants 3 and 4, and this provided descriptive information on the response pattern when both modalities were delivered from the outset.

The tDCS was applied in a bilateral montage, with the anode of the tDCS device (ActivaDose II, ActivaTek Inc., Salt Lake City, UT, USA) placed over the involved hemisphere and the cathode over the unaffected hemisphere. The tDCS device was set to 1 mA for 60 min during gait training, delivered via a pair of sponge electrodes moistened with 0.9% NaCl solution. The exact electrode placement locations were based on a 20-point electrode system, in which we selected two points targeting the motor cortex region corresponding to the left lower limb. Treatments were performed twice weekly over the 8 weeks.

The FES was administered to the tibialis anterior muscle of the impaired lower extremity with one electrode over the common peroneal nerve at the head of the fibula and the other over a motor point in the middle of the muscle belly of the tibialis anterior muscle. The frequency of the FES device (Chattanooga Continuum, DOJ Global, Vista, CA, USA) was set to 35–50 pulses per second (pps), and the pulse duration was 200–300 microseconds, using a symmetrical biphasic current. The parameters were set to produce a visible and functional muscle contraction appropriate for each participant. The heel switch was positioned on the participant’s heel or was adjusted anteriorly to the forefoot region for those participants who made initial contact at the forefoot. The FES was administered for the duration of the gait training (60 min), and its intensity was periodically adjusted to reduce accommodation to the stimulation.

The gait training consisted of treadmill training with forward and backward ambulation, with speed progression as appropriate; ambulation over obstacles; ramp ambulation forward and backward; and stair ambulation up and down. In each condition, cues were to increase toe clearance on the affected limb and achieve heel strike at initial contact. Vitals were collected at the start and end of each session, and heart rate was measured at the end of each activity, along with the Rating of Perceived Exertion (RPE), to determine whether the participant was working at a higher intensity than baseline.

### Follow-up and outcomes

#### Participant 1

**Figure 2:**
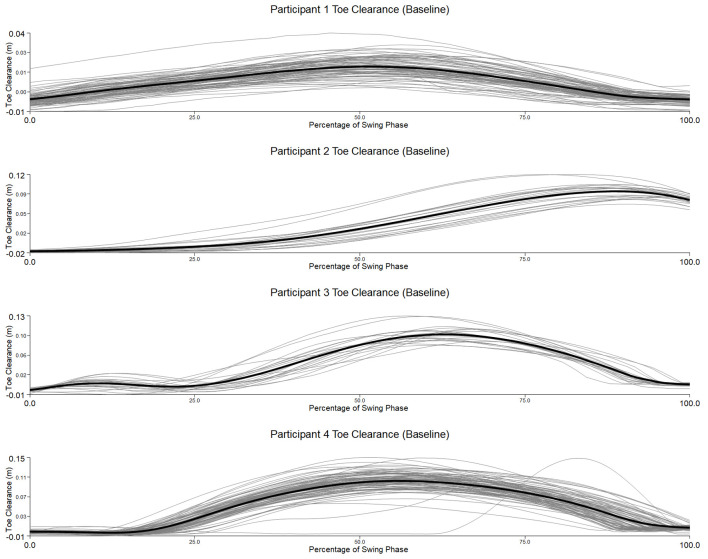
Plot of the toe clearance for participants 1 to 4 (baseline) using the tip virtual marker or digit 5 for participant 1, who was barefoot. The black line represents the mean of the toe clearance pattern from a sample of 10 gait trials of data, out of the 25 collected for each participant. The toe clearance graphs were normalized to 100% of the swing phase.

**Figure 3:**
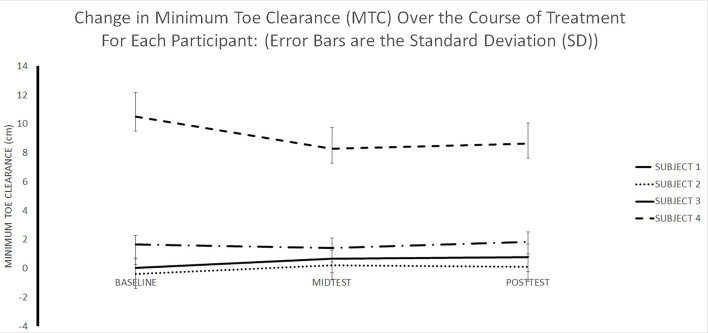
Plot of change in minimal toe clearance subjects 1–4 from baseline through mid-test point to post test.

Participant 1 ambulated with an inverted foot in the swing phase of gait, and the fifth digit was consistently the lowest point. With this gait pattern, there is no MTC event in the mid-swing phase of the gait cycle [52]. That is, after the initial peak after toe-off, the local minimum that typically occurs in mid-swing is no longer present. In fact, the foot was lifted to a maximum point in the mid-swing phase of the gait cycle before being lowered to the floor at initial contact (Fig. 2). At baseline, the clearance was 1.68 ± 0.59 cm; at mid-test, 1.44 ± 0.67 cm; and at post-test, 1.84 ± 0.68 cm (Table 2 and Fig. 3). The average ankle joint angle at this maximum at baseline and the midpoint was −14.72° (SD: 1.54°) and −14.51° (SD: 1.60°), respectively. The ankle joint angle at post-test was −11.42° (SD: 1.44°) during the post-test period, including both tDCS and FES. No improvements were noted in any functional or gait measures (Table 3).

**Table 2:** Minimum or Maximum Toe Clearance and Joint Angle Data at Minimum Toe Clearance for All Four Participants Across All Three Testing Periods

Subject	MTC/MAX L1 (cm) SD	MTC/MAX L2 (cm) SD	MTC/MAX M1 (cm) SD	MTC/MAX M2 (cm) SD	MTC/MAX tip (cm) SD	Mean of lowest MTC values across trials (cm) SD	Percentage of non-MTC for lowest MTC/MAX trials (%)	Ankle joint angle at lowest MTC/MAX (°) SD	Knee joint angle at lowest MTC/MAX (°) SD	Hip joint angle at lowest MTC/MAX (°) SD	Stance hip joint angle at lowest MTC/MAX (°) SD
Baseline											
1	1.68 (0.59)	Non-MTC	Non-MTC	Non-MTC	Non-MTC	1.68 (0.59)	100	−14.72 (1.54)	−28.84 (2.34)	22.75 (1.99)	−6.02 (1.38)
2	−0.26 (0.22)	0.47 (0.43)	Non-MTC	−0.57 (0.28)	Non-MTC	−0.36 (0.63)	14	5.73 (1.35)	−23.15 (1.31)	20.17 (2.25)	5.91 (1.22)
3	0.48 (0.56)	1.86 (0.69)	0.78 (0.060)	1.68 (0.59)	0.04 (0.68)	0.05 (0.67)	1.8	−12.15 (5.51)	−29.24 (3.49)	5.91 (2.36)	−6.80 (1.31)
4	Non-MTC	10.52 (1.68)	Non-MTC	Non-MTC	Non-MTC	10.52 (1.68)	100	−14.34 (1.76)	−19.95 (3.06)	31.45 (2.14)	−1.67 (3.88)
Mid-test (week 4)											
1	1.44 (0.67)	Non-MTC	Non-MTC	Non-MTC	Non-MTC	1.44 (0.67)	100	−14.51 (1.60)	−27.83 (2.23)	20.61 (1.60)	−7.00 (1.67)
2	0.13 (0.66)	1.56 (0.85)	0.07 (0.64)	0.29 (0.62)	−0.25 (0.58)	0.21 (1.04)	46.3	0.65 (2.25)	−51.98 (6.67)	26.07 (4.53)	2.38 (2.83)
3	1.09 (0.70)	1.35 (0.78)	0.67 (0.69)	1.92 (0.70)	1.39 (0.71)	0.68 (0.69)	1.4	−9.88 (4.86)	−28.41 (4.11)	7.94 (2.77)	−3.56 (1.84)
4	Non-MTC	8.30 (1.47)	Non-MTC	Non-MTC	Non-MTC	8.30 (1.47)	100	−16.96 (1.60)	−16.44 (1.90)	25.36 (2.02)	−2.12 (0.96)
Post-test (week 8)											
1	1.84 (0.68)	NON-MTC	Non-MTC	Non-MTC	Non-MTC	1.84 (0.68)	100	−11.42 (1.44)	−27.97 (2.10)	22.39 (2.42)	−0.95 (2.38)
2	0.21 (0.57)	1.42 (0.68)	−0.11 (0.56)	0.11 (0.53)	−0.55 (0.47)	0.12 (0.91)	59.4	0.07 (2.18)	−48.41 (5.80)	26.12 (4.46)	−2.92 (3.48)
3	0.77 (0.77)	1.47 (0.72)	0.98 (0.78)	1.54 (0.73)	0.89 (0.87)	0.82 (0.88)	1.3	−11.38 (6.23)	−29.41 (4.48)	7.81 (2.99)	−5.31 (1.08)
4	Non-MTC	8.65 (1.42)	Non-MTC	Non-MTC	Non-MTC	8.65 (1.42)	100	−14.55 (2.21)	−12.39 (2.24)	20.33 (2.12)	−6.33 (1.38)

Only the point with the lowest mean value is reported when all toe clearances are maximums, and other points are reported as non-MTC. Negative joint angles at the ankle = plantarflexion; knee = flexion; swing hip = extension; and stance hip = abduction. SD: standard deviation; non-MTC: when the toe clearance graph does not have a minimum in the mid-swing phase of the gait cycle; NA: not applicable.

**Table 3:** Functional Outcome and Gait Measures

Subject	FGA	Walk-12	Stroke Impact Scale version 3			ABC scale	Gait speed (m/s) SD	Step length (m) SD	
			Mobility	Participation	Perceived recovery			Left	Right
Baseline									
1	17	60.42	86.1	62.5	75	73.1	0.30 (0.04)	0.28 (0.05)	0.14 (0.05)
2	11	47.92	80.6	68.8	50	65.6	0.75 (0.04)	0.57 (0.03)	0.45 (0.03)
3	24	10.42	**	**	70	95	0.74 (0.05)	0.57 (0.03)	0.57 (0.03)
4	10	31.25	83.3	50	40	34.5	0.18 (0.02)	0.13 (0.02)	0.27 (0.02)
Mid-test (week 4)									
1	16	58.33	86.1	78.1	65	68.1	0.33 (0.04)	0.29 (0.04)	0.18 (0.05)
2	15	29.17	94.4	96.9	55	69.5	0.76 (0.04)	0.59 (0.04)	0.49 (0.04)
3	19	52.08	77.8	34.4	70	76.9	0.67 (0.05)	0.55 (0.03)	0.52 (0.03
4	11	81.25	66.7	56.3	80	56.3	0.21 (0.02)	0.15 (0.03)	0.30 (0.03)
Post-test (week 8)									
1	15	64.58	80.6	71.9	75	68.8	0.30 (0.05)	0.30 (0.06)	0.12 (0.05)
2	17	10.42	94.4	96.9	**	81.3	0.76 (0.05)	0.57 (0.04)	0.50 (0.03)
3	24	45.83	88.9	37.5	75	85	0.73 (0.04)	0.62 (0.03)	0.54 (0.03)
4	13	56.25	72.2	28.1	35	50	0.26 (0.02)	0.17 (0.03)	0.34 (0.02)

**Missing data. Walk-12 formula = Transformed Walk-12 score = [(Raw Walk-12 score − 12)/48] × 100. Stroke Impact Scale subdomains [(Mean item score − 1)/4] × 100. FGA: functional gait assessment; ABC scale: Activities-Specific Balance Confidence Scale.

#### Participant 2

The lowest of the five digitized points that characterized the front of the foot was used to define MTC for each trial. The lowest point from each of the 25 trials was then averaged to determine the mean MTC. The average of these lowest points was −0.36 cm (SD: 0.63 cm) at baseline, 0.21 ± 1.04 cm at mid-test, and 0.12 ± 0.9 cm at post-test (Table 2 and Fig. 3). The tip of the shoe had the lowest average value among the five digitized points in both mid-test and post-test periods, with 100% of trials resulting in non-MTC events at baseline. The average ankle joint angle at the lowest point on the front of the shoe was 5.73° (SD: 1.35°), 0.65° (SD: 2.25°) at mid-test and 0.07° (SD: 2.18°) at post-test. It was also noteworthy that knee flexion (baseline −23.15° (SD: 1.31°); post-test −48.41° (SD: 5.80°)), hip flexion (baseline 20.17° (SD: 2.25°); post-test 26.12° (SD: 4.46°), and hip abduction (baseline 5.91° (SD: 1.22°); post-test −2.92° (SD: 3.48)) increased between baseline and post-test. Between baseline and post-test periods, there was a 6-point improvement in the FGA, a 37.5% reduction in perception of disability on the Walk-12, and a 28.1-point increase in the participation subscale of the stroke impact scale.

#### Participant 3

No single virtual point on the shoe was consistently the lowest point, on average, across all test periods. At baseline, the point at the shoe tip had the lowest average value of 0.05 ± 0.67 cm. At the mid-test and post-test periods, the lowest points were on the medial aspect of the shoe (M1) at 0.68 ± 0.69 cm and on the shoe’s lateral aspect (L1) at 0.82 ± 0.88 cm, respectively. The percentages of non-MTC events at the lowest points in the baseline, mid-test, and post-test periods were 2%, 1.4%, and 5%, respectively. The ankle joint angles at these points at baseline, mid-test, and post-test periods, respectively, were −12.15° (SD: 5.51°), −9.88° (SD: 4.86°), and −11.38° (SD: 6.23°). Concurrently, hip flexion increased from baseline 5.91° (SD: 2.36°) to 7.94° (SD: 2.77°) at mid-test, and remained relatively consistent at post-test 7.81° (SD: 2.99°). The swing phase toe clearance pattern resembled the typical pattern found in healthy adults, with the characteristic two maxima surrounding the minimum of MTC (Fig. 2) [28, 53–55]. However, MTC tended to occur earlier in the swing phase, and the large maximum at the end was not associated with heel strike but occurred before foot-flat. Additionally, there was an increase in the Walk-12 score by 35.41 points and a reduction in the ABC scale of 10 points between baseline and post-test periods (Table 3).

#### Participant 4

The gait pattern did not produce a minimum at MTC. A point on the shoe’s lateral aspect (L2) was consistently the lowest at the maximum. The clearance amounts at the baseline, mid-test, and post-test phases were 10.52 ± 1.68 cm, 8.30 ± 1.47 cm, and 8.65 ± 1.43 cm, respectively (Table 2 and Fig. 3). The average ankle joint angle at this maximum at baseline, mid-test, and post-test phases, respectively, was −14.34° (SD: 1.76°), −16.96° (SD: 1.60°), and −14.55° (SD: 2.21°) (Table 2). These ankle changes were associated with concurrent increased extension in knee (baseline −19.95° (SD: 3.06°); mid-test −16.44° (SD: 1.90°); post-test −12.39° (SD: 2.24°)) and hip (baseline 31.45° (SD: 2.14°); mid-test 25.36° (SD: 2.02°); post-test 20.33° (SD: 2.12°) movements, and stance hip adduction (baseline −1.67° (SD: 3.88°); mid-test −2.12° (SD: 0.96°); post-test −6.33° (SD: 1.38°)). Between the baseline and post-test periods, the Walk-12 score increased by 25 points, and the mobility and participation domains of the Stroke Impact Scale decreased by 11.1 and 21.9 points, respectively (Table 3).

## Discussion

In this case series, we evaluated whether combining FES and tDCS within a PTT session would increase toe clearance at MTC in a cohort of PPS. This increase in toe clearance at MTC was hypothesized to be associated with improved ankle dorsiflexion, potentially indicating improved tibialis anterior (TA) activation. However, the observed changes in MTC could not be interpreted as reflecting ankle dorsiflexion alone. MTC is an endpoint measure influenced by the coordinated motion of multiple joints, and the present kinematic data suggest that participants may have used different multi-joint strategies to modify toe clearance. For example, ankle position did not consistently explain the direction of change in MTC. In some participants, greater plantarflexion would be expected to reduce toe clearance, whereas concurrent changes in hip or knee flexion appeared more consistent with the observed MTC response. Therefore, the hypothesis that increased MTC primarily reflects improved ankle dorsiflexion or enhanced dorsiflexor control is not fully supported by the present data. Further, we did not identify consistent improvements in toe clearance across all participants. This inconsistency was paralleled by the clinical measures, which also showed inconsistent improvements.

Participants 1 and 4 adopted toe clearance strategies that resulted in what Santhiranayagam et al referred to as non-MTC events—or the non-occurrence of a MTC event [52]. The literature provides evidence that non-MTC events occur more frequently in individuals at greater risk of falling [52, 56–59]. Therefore, these participants may employ this strategy to reduce their fall risk. For participant 1, the lateral aspect of her foot (fifth MET) was consistently the lowest point due to ankle inversion during the swing phase. With this non-MTC strategy, the toe clearance curves peaked in the midswing phase, where MTC typically occurred [6, 27, 60]. The toe clearance values observed at this peak (baseline: 1.68 cm ± 0.59; mid-test: 1.44 cm ± 0.67 cm, and post-test: 1.84 ± 0.68 cm; Table 2) were comparable to the mean MTC values reported in the literature for both healthy young and older adults, which typically range from 1 to 3 cm [6, 27]. Also, there was slightly more dorsiflexion (post-test: −11.42 ± 1.44°) at the post-test with the inclusion of both tDCS and FES than at the mid-test (−14.51± 1.60°), where only tDCS was included. In fact, at mid-test, ankle dorsiflexion remained relatively unchanged from baseline (−14.72 ± 1.54°). These changes in toe clearance were not reflected in functional measures, as they were not accompanied by substantive changes in gait metrics or the improvement in the participant’s perceptions of their gait ability. It is possible that these changes in MTC may not be reflected in broader functional measures, as MTC may be a specific variable of the swing phase that may have implications for trip-related falls.

For participant 4, who received tDCS, FES, and PTT beginning at baseline, the maximum non-MTC event count was much higher than for participant 1. This higher clearance may be associated with a greater metabolic cost, but this remains unclear, as increasing MTC by 4 cm, which is less than the 5 to 7 cm increase observed for this participant, did not significantly affect dorsiflexion moments and muscle forces [61]. The maximum in the toe clearance curve decreased between baseline (10.52 ± 1.68 cm) and post-test (8.65 ± 1.42 cm). Ankle position did not consistently explain the observed changes in MTC. For example, ankle plantarflexion increased at mid-test relative to baseline, which would be expected to reduce toe clearance and was consistent with the lower MTC observed at that time point. However, ankle position returned toward baseline at post-test while toe clearance remained lower, suggesting that ankle motion alone did not account for the MTC response. The concurrent increases in knee and hip extension suggest that proximal joint motion may have contributed to the reduced toe clearance in this participant. These changes were not associated with improvements in functional measures, reiterating the possibility that changes in MTC may not translate to consistent functional improvements. Interestingly, neither of these participants (participants 1 and 4) who used this non-MTC strategy achieved isolated active dorsiflexion.

Participants 2 and 3 achieved active dorsiflexion, although weak (grade 2 manual muscle test score), and had MTC events. Participant 2, who began with FES alone, noted an increase in the mean lowest MTC amount from −0.36 ± 0.63 cm at baseline to 0.21 ± 1.04 cm at mid-test. However, with the addition of tDCS at mid-test, there was a slight reduction in the average lowest MTC at post-test to 0.12 ± 0.90 cm. Further, the slight increase in toe clearance amount from baseline to mid-test was not accompanied by an increase in dorsiflexion as expected [24, 47, 51, 56, 62, 63] but a decrease from 5.73 ± 1.35° to 0.65° (2.25°) at the lowest average point. The lower toe clearance amount at MTC at post-test, relative to mid-test, corresponded with slightly less dorsiflexion (0.07 ± 2.18°). A combination of increased hip flexion of the swing limb and hip abduction of the stance limb may have offset the lower dorsiflexion, accounting for the higher clearances at mid-test. Therefore, these findings suggest that higher toe clearance should not be assumed to result solely from increased ankle dorsiflexion. Additionally, there were clinically meaningful improvements in the FGA from baseline to post-test (a 6-point increase) and a 37% reduction in their perceived walking limitations on the Walk-12, shifting their perception from moderate to mild. These findings may reflect concurrent functional improvements in this patient unrelated to changes in MTC, as functional measures may capture broader domains, including balance, strength, endurance, and overall mobility, whereas MTC may be a specific swing-phase variable. It is unclear from this study why improvements in MTC in this particular patient were associated with concurrent functional improvements, and this should be explored in other studies.

For participant 3, the improvement in the average lowest toe clearance at MTC was more dramatic, increasing from 0.05 ± 0.67 cm at baseline to 0.68 ± 0.69 cm at mid-test, then to 0.82 ± 0.88 cm at post-test. However, the expectation that this improvement in the average toe clearance amount at MTC would be consistently accompanied by increased dorsiflexion was not realized [24, 47, 51, 56, 62, 63]. “In fact, there was an increase in relative dorsiflexion between baseline and mid-test periods, going from −12.15° (SD: 5.51°) to −9.88° (SD: 4.86°) at mid-test, as expected, but there was relatively more plantarflexion at post-test (−11.38 ± 6.23°) than at mid-test. Because MTC is an endpoint measure that can be influenced by the individual or coordinated kinematics of multiple lower-limb joints, the present data suggest that the slightly greater hip flexion at mid-test and post-test relative to baseline may have contributed to the increases in MTC for participant 3. However, the authors cannot definitively determine whether the observed changes in MTC in participant 3 and the other three participants were primarily related to ankle dorsiflexion or to changes in other lower-limb joints [51]. Therefore, it remains possible that different participants may use distinct multi-joint strategies to modify toe clearance, including proximal compensations involving the hip, knee, and pelvis. These changes, while notable for MTC, did not translate into improvements on either the FGA or the Walk-12 scales, and the ABC scale did exceed the minimal detectable change. These findings suggest that improvements in MTC may not consistently correspond with measurable functional gains or improved perceptions of walking limitations in this population. However, because MTC is more directly related to foot clearance and may reflect trip risk, changes in MTC may not be fully captured by broader functional outcome measures.

Interestingly, for participants 2 and 3, non-MTC events were frequent on the part of the shoe, with the lowest average clearance when toe clearances were small. The mean increase in toe clearance at MTC for these participants between baseline and post-test was ((0.12 – (−0.36)) + (0.82 – 0.05))/2 = (0.48 + 0.77)/2 = 0.63 cm. This mean increase exceeds the previously reported error estimate of ± 0.2 cm for virtual point-based foot clearance calculations [64]. At the individual level, both participants’ changes were also beyond this measurement error range. These findings suggest that the observed increases in foot clearance were likely detectable changes rather than measurement variability alone. However, because a minimal clinically important difference has not been established for MTC, and because this study did not directly measure trip or fall incidence, these changes cannot be interpreted as definitive evidence of reduced fall risk. Further research is needed to determine whether changes of this magnitude are clinically meaningful. It was unclear from these participants whether including tDCS was beneficial, as participant 2 showed improvement with FES alone, and although participant 3’s improvement was larger, there was no FES-only control condition for participant 3. Therefore, it is impossible to determine whether tDCS provided additional benefits over FES.

Interestingly, all participants without active dorsiflexion produced all non-MTC events. In contrast, participants with MTC events tended to produce non-MTC events more frequently in the front foot areas closest to the ground. For example, for participant 2, the shoe tip was closest to the ground during the mid-test and post-test periods. Consequently, with more non-MTC events, this area contributed less to the lowest average toe clearance at MTC, thereby increasing the lowest average toe clearance because these were no longer the minimum events to be counted. Based on this analysis, using a single point at the tip of the shoe or a single marker, a common practice utilized in MTC studies [28, 61, 65–68], would not be sufficient to characterize the complexity displayed by these participants with chronic stroke in achieving toe clearance.

The pattern of change in the clinical outcome measures was also unclear. For participants 1 and 4, only participant 4 noted clinically significant improvements in the functional measures on the Walk-12 [69] and the mobility [70] and participation [71] subdomains of the Stroke Impact Scale, despite not experiencing changes in toe clearance. For participants 2 and 3, who noted improvements in toe clearance at MTC, participant 2 showed significant improvements on the functional measures of the FGA [72], Walk-12 [69], and the participation subscale of the Stroke Impact Scale [71]. For participant 3, there was an unanticipated worsening in perceived disability, as evidenced by an increase in the Walk-12 score, and the ABC scale did not achieve a clinically meaningful improvement despite improved toe clearance [73].

There were limitations associated with this study. First, participant 3 wore different shoes during the mid-test session, and the shoe type has been shown to affect MTC magnitude [74, 75]. However, the identical shoe to the baseline was used at the post-test. Second, electromyographic data were not collected from the tibialis anterior muscle, so we cannot infer whether any of the improvements were due to improved tibialis anterior activation. Furthermore, any of the six lower-limb joints may increase toe clearance at MTC, further confounding the question of whether increases in toe clearance in this present study were related to increased dorsiflexion resulting from improved TA activation. Third, negative values were recorded in this study, which is an artifact introduced by measurement error but is not atypical in MTC studies [47, 64, 76, 77]. Fourth, we did not perform an inter-rater reliability assessment of the raters for the clinical outcomes. Fifth, the heterogeneous intervention structure should be interpreted as an exploratory case series design rather than a comparative efficacy design. Specifically, the design permits examination of within participant response patterns before and after the addition of a second modality, but it does not isolate the independent effect of tDCS, FES, or physical therapy training, as there was no independent comparator condition. Therefore, improvements observed during the combined treatment phase may reflect the added modality, continued response to the initial modality, cumulative treatment exposure, repeated task practice, physical therapy training, or an interaction among these components. Accordingly, the findings should be interpreted as hypothesis generating evidence regarding whether combined tDCS and FES may provide added benefit in individual cases, rather than definitive evidence that the combined intervention is superior to either modality alone.

### Learning points

We noted an average MTC increase of 0.63 cm in PPS who achieved active dorsiflexion at the outset of treatment, but no improvement in those who did not. While the increase in toe clearance exceeded the measurement error, it cannot be interpreted as clinically significant, as neither a minimal clinically important difference nor another clinically meaningful threshold has been established. We anticipated a correlation between MTC improvements and ankle dorsiflexion, but results were inconsistent and often concurrent with changes in proximal joints. Furthermore, since tibialis anterior muscle activation was not measured, its role remains unclear. The MTC improvements were not consistently associated with functional gains or patient-reported walking limitations, suggesting that MTC may indicate trip risk more than broader functional changes. Lastly, because we did not isolate the effects of tDCS, FES, or PTT, these findings are hypothesis generating rather than definitive proof of their combined superiority.

## Data Availability

The data supporting the findings of this study are available from the corresponding author upon reasonable request.

## Abbreviations

ABC scale: Activity-specific Based Confidence scale
AFOs: ankle-foot orthoses
ASIS: anterior superior iliac spines
CAST: calibrated anatomical system technique
CVA: cerebrovascular accident
FES: functional electrical stimulation
MET: fifth metatarsal
MTC: minimum toe clearance
pps: pulses per second
PPS: persons post-stroke
PTT: physical therapy training
RPE: Rating of Perceived Exertion
TA: tibialis anterior
tDCS: transcranial direct current stimulation
